# LaAlO_3_:Mn^4+^ as Near-Infrared Emitting Persistent Luminescence Phosphor for Medical Imaging: A Charge Compensation Study

**DOI:** 10.3390/ma10121422

**Published:** 2017-12-12

**Authors:** Jiaren Du, Olivier Q. De Clercq, Katleen Korthout, Dirk Poelman

**Affiliations:** LumiLab, Department of Solid State Sciences, Ghent University, Krijgslaan 281-S1, 9000 Ghent, Belgium; Jiaren.Du@ugent.be (J.D.); Olivier.DeClercq@ugent.be (O.Q.D.C.); Katleen.Korthout@ugent.be (K.K.)

**Keywords:** persistent luminescence, Mn^4+^-activated phosphors, charge compensation, LaAlO_3_:Mn^4+^, solid-state reaction

## Abstract

Mn^4+^-activated phosphors are emerging as a novel class of deep red/near-infrared emitting persistent luminescence materials for medical imaging as a promising alternative to Cr^3+^-doped nanomaterials. Currently, it remains a challenge to improve the afterglow and photoluminescence properties of these phosphors through a traditional high-temperature solid-state reaction method in air. Herein we propose a charge compensation strategy for enhancing the photoluminescence and afterglow performance of Mn^4+^-activated LaAlO_3_ phosphors. LaAlO_3_:Mn^4+^ (LAO:Mn^4+^) was synthesized by high-temperature solid-state reaction in air. The charge compensation strategies for LaAlO_3_:Mn^4+^ phosphors were systematically discussed. Interestingly, Cl^−^/Na^+^/Ca^2+^/Sr^2+^/Ba^2+^/Ge^4+^ co-dopants were all found to be beneficial for enhancing LaAlO_3_:Mn^4+^ luminescence and afterglow intensity. This strategy shows great promise and opens up new avenues for the exploration of more promising near-infrared emitting long persistent phosphors for medical imaging.

## 1. Introduction

Persistent luminescence materials relate to a particular optical phenomenon whereby the light emission can last for several hours after the excitation has stopped [1,2,3]. The basic principles and physics behind persistent luminescence materials are related to two kinds of active centers: traps and emitters. Emitters release light in the wavelength range of interest and traps contribute to the duration time of the long persistent phosphor. The latter are believed to originate from lattice defects, co-dopants or impurities. The persistent luminescence phenomenon can be related to an optical battery as discussed in other papers [4].

The past two decades have witnessed rapid development and enormous advances to establish persistent luminescence materials for various applications including night-vision, emergency route illumination, security signs, traffic night signage, dials, decorative objects and toys [5]. Representative examples are SrAl_2_O_4_:Eu^2+^,Dy^3+^ (green emission) [1] and CaAl_2_O_4_:Eu^2+^,Nd^3+^ (blue emission) [6]. A pioneer proof-of-concept work published in 2007 first realized the application of in vivo imaging by using the near-infrared persistent luminescent material Ca_0.2_Zn_0.9_Mg_0.9_Si_2_O_6_:Eu^2+^,Dy^3+^,Mn^2+^ as biomarker, hereby opening up new avenues for the widespread uses of persistent luminescent phosphors [7]. As for the use of in vivo imaging, the emitting wavelength of the phosphor is required to be located in the biological optical window (i.e., the first near-infrared window in the wavelength between 650 nm and 950 nm or the second near-infrared window in the wavelength between 1000 nm and 1350 nm). In these wavelength ranges, scattering, absorption and auto-fluorescence are limited and biological tissue is partly transparent [8]. Thus, the development of deep red/near-infrared emitting persistent phosphors has attracted much attention for application to in vivo bio-imaging systems or medical imaging.

Near-infrared emitting persistent phosphors have several advantages in comparison with other optically active particles, such as quantum dots and upconversion nanoparticles. Near-infrared quantum dots are limited by their potential toxicity and upconversion luminescent particles are hindered by the need for high energy lasers, which can lead to tissue damage [9,10]. Autofluorescence, one of the main drawbacks encountered with classic imaging probes, can be greatly reduced by using the near-infrared emitting persistent probes. Before its injection, the probe is well pre-excited outside the body of small animals to avoid autofluorescence. The signal to background ratio can be enhanced and local heating effects coming from high-power laser excitation can be avoided. Toxic effects can also be reduced when choosing appropriate phosphors, although long-term cytotoxicity studies still need to be undertaken [11]. Efforts to work on persistent luminescent nanoprobes for in vivo bioimaging applications were made, providing highly sensitive optical detection from living tissues and showing promising prospects in future practical use [12].

Traditionally, Cr^3+^-doped deep red/near-infrared emitting persistent luminescence nanoparticles (PLNPs) are used as the key phosphors for in vivo imaging in small animals. Many Cr^3+^-doped phosphors are widely investigated, such as ZnGa_2_O_4_:Cr^3+^ [13], Zn_3_Ga_2_Ge_2_O_10_:Cr^3+^ [14], Zn_3_Ga_2_SnO_8_:Cr^3+^ [15] and LiGa_5_O_8_:Cr^3+^ [16,17,18]. Currently, manganese doped compounds, especially Mn^4+^-activated phosphors, are considered as a promising alternative to Cr^3+^-doped nanomaterials [19]. The tetravalent manganese ions (Mn^4+^) can be doped in both fluorides and oxides [20,21,22,23]. Great effort has been made to the development of Mn^4+^-activated oxide compounds. However, it remains a challenge to improve Mn^4+^-activated phosphors with long afterglow and strong luminescence intensity compared with traditional Cr^3+^-doped nanocrystals. This problem can be caused by the difficulty of stabilizing the Mn ions in the correct oxidation state due to the charge imbalance.

In the present work, manganese doped perovskite lanthanide aluminates (LaAlO_3_) were synthesized by a high-temperature solid-state reaction method, similar to other reported work [19,24,25] and the persistent luminescence behavior was optimized. In order to improve the near-infrared persistent luminescence intensity of LaAlO_3_:Mn^4+^ phosphors, a series of samples were prepared using solid state reaction in air through co-doping with a variety of ions with different valence states. The doping effects and their possible mechanisms were investigated. The afterglow and luminescence intensity of this material can be strongly improved by this strategy of co-doping, leading to charge compensation or the introduction of new trap levels.

## 2. Results and Discussion

### 2.1. Crystal Structure

**Figure 1:**
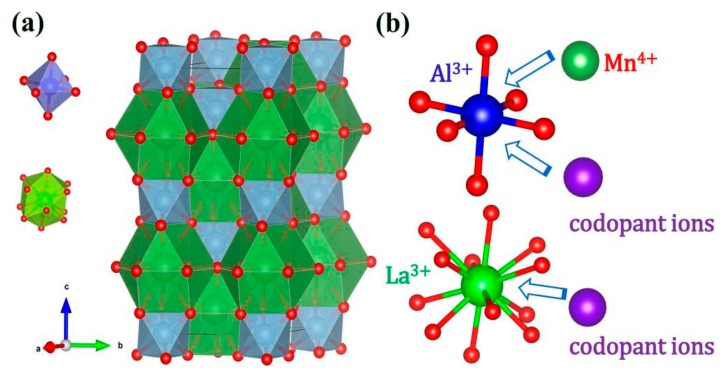
(**a**) Crystal structure of LaAlO_3_ (blue unit is AlO_6_ octahedron and green unit is LaO_12_ polyhedron); (**b**) Doping positions for Mn^4+^ and other dopant ions, the Mn^4+^ ion can occupy Al^3+^ ion site in the AlO_6_ octahedral unit.

Figure 1a shows the crystal structure of LaAlO_3_ drawn on the basis of the Inorganic Crystal Structure Database (ICSD No. 153821). LaAlO_3_ is described in the trigonal crystal system with space group R-3cH (space group number 167) and lattice parameters a = 5.3598 Å, b = 5.3598 Å, c = 13.086 Å, Volume = 325.56 Å^3^ and z = 6 [26]. As is shown in Figure 1a, the crystal structure corresponds to the rhombohedral, nearly cubic perovskite structure, which involves a rotation of the AlO_6_ octahedra with respect to cubic perovskite as reported elsewhere [27,28]. There are two types of units in the crystal structure: AlO_6_ octahedra and LaO_12_ polyhedra. The central Al^3+^ cation is in 6-fold oxygen coordination and forms AlO_6_ octahedral units (blue unit in Figure 1). The La^3+^ cations are located in a polyhedral unit with 12-fold oxygen coordination (green unit in Figure 1). It is reported that the La-sites have D_3_ point symmetry [29] and Cr^3+^-doped LAO confirm a C_3i_ symmetry for the Al site [30]. Both cation sites thus have reduced symmetry from pure O_h_ symmetry, which results from a contraction along and a small rotation of AlO_6_ octahedra around the c-axis of the LAO host. However, an inversion center is maintained for the Al-site. The reduction from pure octahedral symmetry is expected to result in a small splitting of the ^2^E level and thus the zero-phonon transition of Mn^4+^. The ionic radius of the Mn^4+^ ion, Al^3+^ ion and La^3+^ ion is 53 pm, 53.5 pm and 136 pm in 6-fold octahedral coordination (Mn^4+^ ion, Al^3+^ ion) and 12-fold coordination (La^3+^ ion), respectively [31]. It is well known that the Mn^4+^ ion usually stabilizes in an octahedral site with 6-fold coordination [21], thus, Mn^4+^ ions will supposedly occupy the Al^3+^ ion sites in the LaAlO_3_ host as shown in Figure 1b. The similar ionic radius helps the substitution between dopant Mn^4+^ ion and central Al^3+^ ion in the AlO_6_ octahedra.

**Figure 2:**
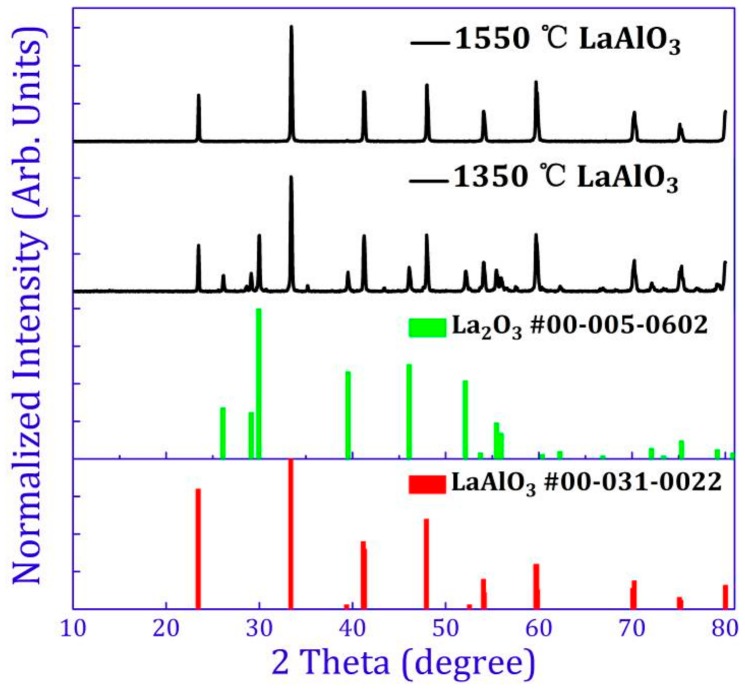
A comparison of XRD (X-ray diffraction) patterns between 1350 °C and 1550 °C. The impurity phase in the XRD pattern at 1350 °C is assigned to La_2_O_3_ and the optimized temperature for synthesizing is 1550 °C. The standard XRD data of LaAlO_3_ and La_2_O_3_ are illustrated in red and green bars respectively. The intensities of XRD patterns are normalized to arbitrary units [0,1].

XRD (X-ray diffraction) patterns of un-doped LaAlO_3_ sintered at 1350 °C, 1400 °C, 1450 °C, 1500 °C, 1550 °C, 1600 °C and 1650 °C are shown in Figure S1. At higher sintering temperatures (above 1550 °C), the XRD patterns of these samples match well with the standard XRD data of LaAlO_3_ (No. 00-031-0022). At lower sintering temperatures, especially at 1350 °C, a different pattern is displayed. XRD patterns of samples synthesized at 1350 °C and 1550 °C are compared in Figure 2. The impurity phase at 1350 °C is found to be La_2_O_3_ (as shown in Figure 2) and the optimized temperature for synthesizing LaAlO_3_ was chosen at 1550 °C. The standard XRD data of LaAlO_3_ (No. 00-031-0022) and La_2_O_3_ (No. 00-005-0602) are illustrated in red and green bars respectively in Figure 2. Detailed XRD patterns of the obtained LaAlO_3_ phosphor with different concentrations of Mn^4+^ and Mn^4+^,R sintered at 1550 °C demonstrate that doping of Mn^4+^ ions or Mn^4+^,R (R = Ge^4+^, Si^4+^, Ti^4+^, Zr^4+^, Ba^2+^, Ca^2+^, Mg^2+^, Sr^2+^, Cl^−^, Li^+^, Na^+^) co-dopants does not cause any significant structural changes of the LaAlO_3_ host for any of the dopants (XRD patterns not shown). Energy-dispersive X-ray (EDS) mapping indicates the homogeneous distribution of manganese in the host (not shown). In agreement with the previous reports, doping of manganese ions is perfectly feasible in the LaAlO_3_ host [19,32].

### 2.2. Luminescence Properties

**Figure 3:**
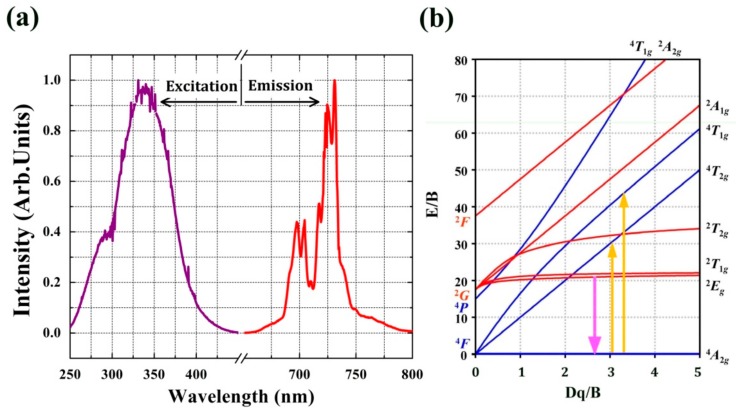
(**a**) Photoluminescence (PL) and photoluminescence excitation (PLE) spectra of LaAlO_3_:0.5%Mn^4+^phosphor. PL and PLE spectra (λ_ex_ = 335 nm and λ_em_ = 731 nm) are in the range 250–450 nm and 650–800 nm, respectively; (**b**)Tanabe-Sugano energy level diagram of a d^3^ configuration (Mn^4+^ion in the octahedron).

**Figure 4:**
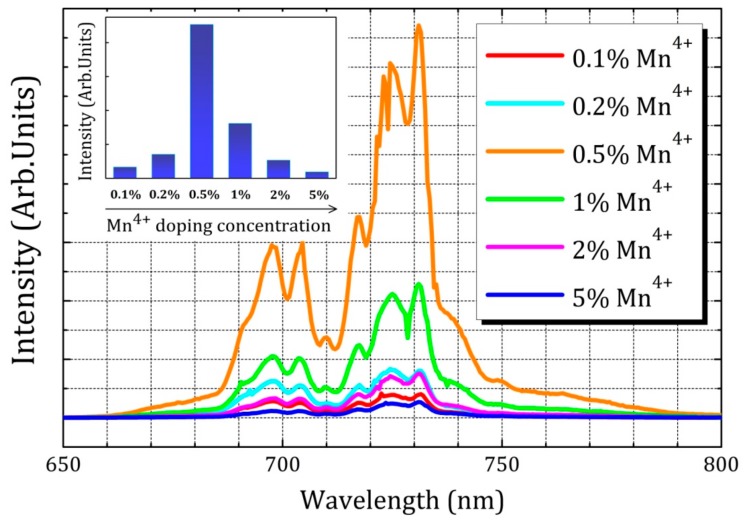
A comparison of photoluminescence (PL) spectra of LaAlO_3_:0.1%Mn^4+^, LaAlO_3_:0.2%Mn^4+^, LaAlO_3_:0.5%Mn^4+^, LaAlO_3_:1%Mn^4+^, LaAlO_3_:2%Mn^4+^ and LaAlO_3_:5%Mn^4+^ phosphors. The emission spectrum is acquired under 335 nm excitation. The inset gives the relative emission intensity while increasing Mn^4+^ doping concentration from 0.1% to 5%.

The room-temperature photoluminescence (PL) spectrum of LaAlO_3_:0.5%Mn^4+^ phosphor upon excitation at 335 nm exhibits narrow emission bands in the range 650–800 nm due to the ^2^E_g_→^4^A_2g_ spin-forbidden transitions in Mn^4+^ ions, with a maximum located at 731 nm as shown in Figure 3a. The spectrum consists of several sharp features, peaking at (from left to right) 697.5 nm, 704.5 nm, 710.5 nm, 718 nm, 724.5 nm and 731 nm, corresponding to the spin-forbidden ^2^E_g_→^4^A_2g_ transition and the vibrational sidebands of zero-phonon line (ZPL) with phonon assistance. Due to its high effective positive charge, Mn^4+^ experiences a large crystal field and hence no transitions from the ^4^T_2_ level are expected, in contrast to Cr^3+^-based phosphors [17]. The zero-phonon line (ZPL) is located at 718 nm in Figure 3. The ZPL is surrounded by both anti-Stokes and Stokes phonon side bands. The ZPL here (718 nm, ~13927 cm^−1^) has a larger wavelength than the value of 712 nm (~14045 cm^−1^), measured at 300 K and reported by Van Ipenburg et al. [33] but corresponds to the value reported by Li et al. [19]. An assignment of the sidebands to the type of vibration was done by Van Ipenburg et al. [33] and the anti-Stokes sidebands at 697.5 nm, 704.5 nm and 710.5 nm appear in our sample. The ^2^E_g_→^4^A_2g_ transition has a small electron-phonon coupling and the excited ions usually relax non-radiatively to ^2^E_g_ followed by the spin-forbidden ^2^E_g_→^4^A_2g_ transition, thus resulting in narrow-band emission lines. This is in contrast with the broad bands in the excitation spectrum that correspond to spin-allowed ^4^A_2_→^4^T_1_ and ^4^A_2_→^4^T_2_ transitions, with larger electron-phonon coupling [23,34]. It has been widely reported that the spectra of Mn^4+^ ions exhibit a combination of broadband excitation bands and sharp emission lines [34,35]. Usually, the excitation and emission peaks of Mn^4+^ ions in many other oxide hosts are observed around 300 nm and above 650 nm, respectively [36,37,38]. This PL behavior proves that Mn is indeed incorporated in the LaAlO_3_ lattice and is incorporated in a 4+ oxidation state, since Mn^2+^ is expected to show an entirely different and broad emission spectrum. Mostly, the excitation spectrum of Mn^2+^ is very characteristic for the d^5^ electron configuration. The photoluminescence excitation (PLE) spectrum of the phosphor at room temperature is also shown in Figure 3a. The PLE spectrum (λ_em_ = 731 nm) shows a broad band with the main peak located at 335 nm and ranging from 250 nm to 450 nm. The broad PLE band is mainly attributed to ^4^A_2_→^4^T_1_ and ^4^A_2_→^4^T_2_ transitions of Mn^4+^ ion as illustrated in the Tanabe-Sugano energy level diagram for 3d^3^ ions [39]. When Mn^4+^ ions are situated in the LaAlO_3_ host with octahedral coordination, the dependence of energy levels of Mn^4+^ on crystal field strength can be clearly illustrated by the Tanabe-Sugano energy level diagram in Figure 3b. A comparison of PL spectra of LaAlO_3_:0.1%Mn^4+^, LaAlO_3_:0.2%Mn^4^, LaAlO_3_:0.5%Mn^4+^, LaAlO_3_:1%Mn^4+^, LaAlO_3_:2%Mn^4+^ and LaAlO_3_:5%Mn^4+^ is illustrated in Figure 4. The PL intensity of LaAlO_3_:Mn^4+^ phosphors increases with increasing Mn^4+^ ion concentration within the range 0.1% to 0.5% and decreases upon further increasing Mn^4+^ ion concentration from 0.5% to 5%. The former effects are presumably due to the effective Mn^4+^ concentration, which dominatingly determines PL intensity in this host. When the content of the doping Mn^4+^ ions is relatively low, the effective Mn^4+^ concentration is approximately proportional to the content of the doping Mn^4+^ ions. Thus, increasing the content of Mn^4+^ ions from 0.1% to 0.5%, the PL intensity of LaAlO_3_:Mn^4+^ phosphors increases synchronously. The latter observation could be attributed to the concentration quenching phenomenon of Mn^4+^ ions. It can be seen that the optimal doping concentration of Mn^4+^ ions is 0.5%.

**Figure 5:**
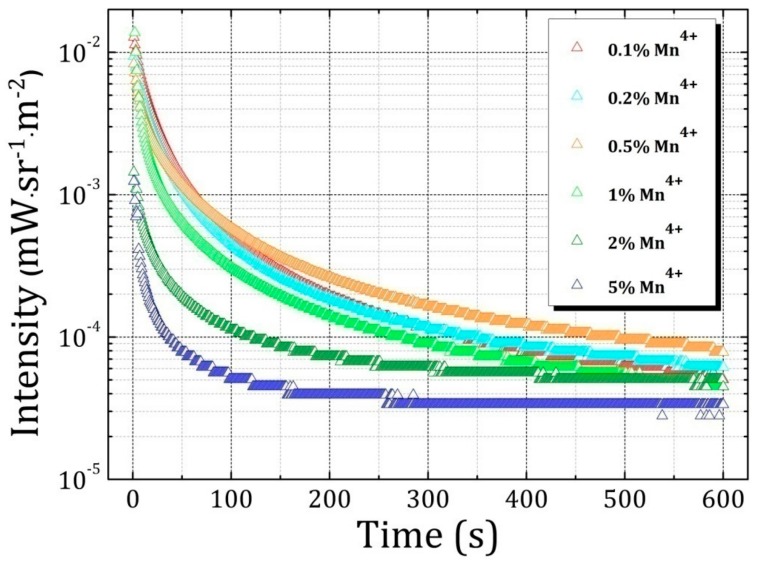
Persistent luminescence decay curves of Mn^4+^-doped LaAlO_3_ phosphors after 5 min of irradiation with a Xenon arc lamp. The concentrations of Mn^4+^ions in LaAlO_3_host are 0.1%, 0.2%, 0.5%, 1%, 2%, 5% as shown in different colors.

Figure 5 shows the persistent luminescence decay curves of LaAlO_3_:0.1%Mn^4+^, LaAlO_3_:0.2%Mn^4+^, LaAlO_3_:0.5%Mn^4+^, LaAlO_3_:1%Mn^4+^, LaAlO_3_:2%Mn^4+^, LaAlO_3_:5%Mn^4+^ phosphors after 5 min of irradiation with a Xenon arc lamp. It was also found that 0.5% Mn^4+^ is also the optimal doping concentration for the afterglow intensity and duration.

### 2.3. Charge Compensation Strategy

**Figure 6:**
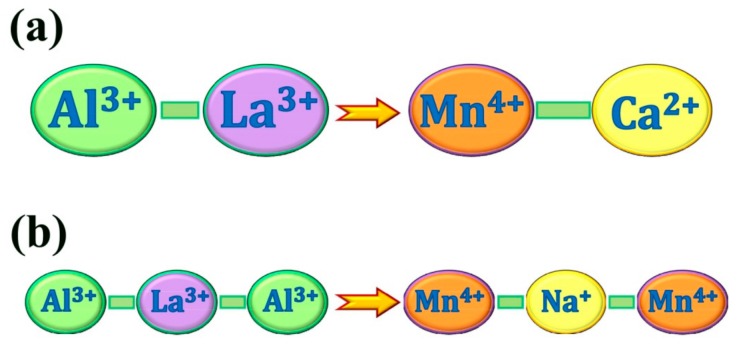
A schematic of charge compensation strategies for LaAlO_3_:Mn^4+^ phosphor. (**a**) The charge compensation for the unit of Al^3+^-La^3+^ and Mn^4+^-Ca^2+^; (**b**) The charge compensation for the unit of Al^3+^-La^3+^-Al^3+^ and Mn^4+^-Na^+^-Mn^4+^.

To enhance the photoluminescence and afterglow performance of Mn^4+^-activated LaAlO_3_ phosphor, a charge compensation strategy was proposed. As mentioned before, Mn^4+^ ions are supposed to substitute the Al^3+^ ions in the LaAlO_3_ host. Thus, a charge imbalance occurs when the substitution happens. With the aid of lower valence state ions to balance the superfluous positive charge of tetravalent manganese, charge compensation is expected. A schematic of charge compensation strategies is illustrated in Figure 6 for LaAlO_3_:Mn^4+^ phosphor. For the divalent cations such as Ca^2+^ in Figure 6a, the unit of Mn^4+^-Ca^2+^ can be selected as a substitutable alternative to the unit of Al^3+^-La^3+^ to the charge compensation. As for the monovalent cations (for example, Na^+^ in Figure 6b), the units of Mn^4+^-Na^+^-Mn^4+^ and Al^3+^-La^3+^-Al^3+^ are equivalent in number of charges. We expect Mn^4+^ to substitute for Al^3+^ and Ca^2+^ to substitute for La^3+^ based on the following considerations: Ionic radii, coordinated environment and chemical stability. For chemical stability, it is well known that the Mn^4+^ ion usually stabilizes in an octahedral site with 6-fold coordination (in Al^3+^ site). Mn^4+^ ion could hardly be situated in 12-fold coordinated environment (La^3+^ site). In an octahedral environment (in Al^3+^ site), the Mn-3d states are split into three- and two-fold degenerate t_2g_ and e_g_ states, respectively. The three Mn-3d electrons of Mn^4+^ exactly fill the majority-spin t_2g_ states. The crystal field splitting creates a large gap between the t_2g_ and e_g_ states, stabilizing the 4+ oxidation state [21,40,41]. Therefore, Mn^4+^ ions are usually found on octahedral sites of solids [40,41]. For Ge^4+^ and Ti^4+^, they also have a stable chemical environment like the Mn^4+^ ion, so we adopted these ions as co-dopants.

**Figure 7:**
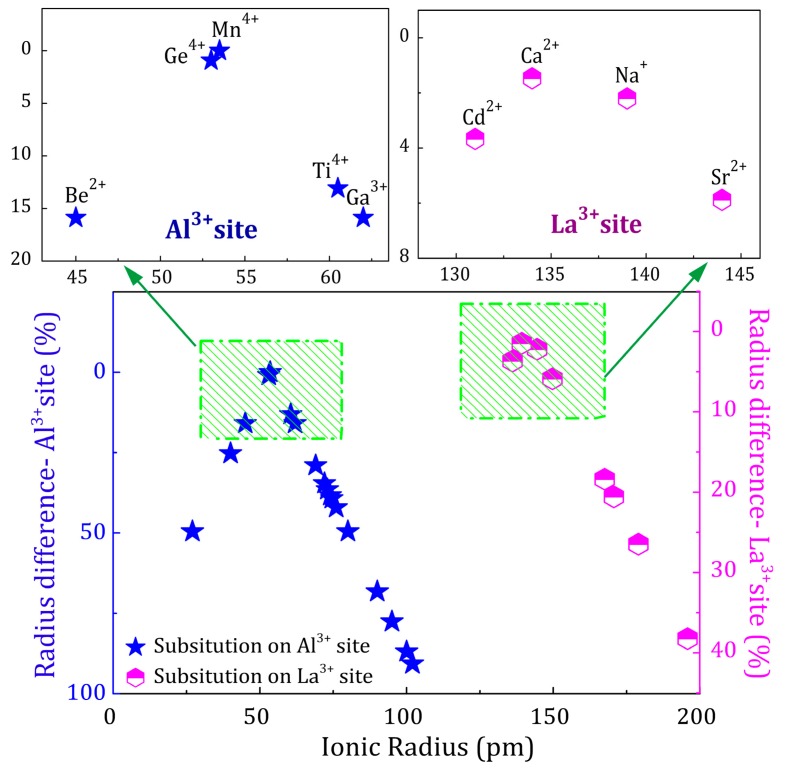
Radius difference between doping ions and substitutable central ions. Blue stars are supposed to substitute on Al^3+^ site with coordination number VI and half-filled pink hexagons are supposed to substitute on La^3+^ site with coordination number XII. Stars and hexagons located in the green wireframe are feasible candidates for doping.

Suitable charge-compensating co-dopants should fulfill certain requirements. The difference in ionic radius between doping ions and central ions plays a critical role in possible substitution and consummate incorporation. The substitution of ions in a crystal lattice has been discussed in depth [42]. It is possible to replace an ion in a specific lattice position with a dopant and not disturb the crystal structure when both ions differ in size by no more than a certain radius ratio for a given coordination number, according to the work of Linus Pauling [42]. It is expected that a smaller radius difference (no more than 15% or 20%) leads to a better substitution. 6-fold coordinated Al^3+^ ion has an ionic radius of 53.5 pm and 12-fold coordinated La^3+^ ion has an ionic radius of 136 pm. Figure 7 shows the radius difference between possible doping ions and central ions [31]. Ions in blue stars are supposed to substitute on the Al^3+^ site with coordination number VI and ions while half-filled pink hexagons are supposed to substitute on the La^3+^ site with coordination number XII (details in Tables S1 and S2). Both tetravalent cations and lower valence state ions are selected for comparison. Stars and hexagons located in the green wireframe are feasible candidates for doping, considering the similarity in ionic radius and coordinated environment in the host. In the case of LaAlO_3_, Ge^4+^ ions and Na^+^, Ca^2+^, Sr^2+^ ions are located in the two green wireframes. Ge^4+^ ions are supposed to replace Al^3+^ ions, while Na^+^, Ca^2+^ and Sr^2+^ ions are substitutable for La^3+^ ions (shown in Figure 6).

### 2.4. Influence of Various Co-Dopants

To obtain systematic information on the influence of other dopants, various kinds of ions can be taken into account. Some other research groups also found that tetravalent cations (such as Ge^4+^) or negative charge ions (such as Cl^−^) could be added as co-dopants in CaAl_12_O_19_ and SrMgAl_10_O_17_ hosts [43,44,45]. Thus, tetravalent cations (Ge^4+^, Si^4+^, Ti^4+^, Zr^4+^), divalent cations (Ba^2+^, Ca^2+^, Mg^2+^, Sr^2+^), monovalent cations (Li^+^, Na^+^) and negative charge ions (Cl^−^) were all selected as discussed above. A series of 2%Mn^4+^,2%R (R = Ge^4+^, Si^4+^, Ti^4+^, Zr^4+^, Ba^2+^, Ca^2+^, Mg^2+^, Sr^2+^, Li^+^, Na^+^, Cl^−^) co-doped phosphors were synthesized at 1550 °C and PL measurements were performed at room temperature. PL spectra of 2%Mn^4+^,2%R (R = Ge^4+^, Si^4+^, Ti^4+^, Zr^4+^, Ba^2+^, Ca^2+^, Mg^2+^, Sr^2+^, Li^+^, Na^+^, Cl^−^) phosphors are shown in Figures S2–S4. Some co-dopants such as Li^+^, Mg^2+^ and Si^4+^ ions did not contribute much to the enhancement of PL intensity. Interestingly, it was found that co-doping with R (R = Na^+^, Cl^−^, Ge^4+^, Ca^2+^, Sr^2+^ and Ba^2+^) increases the PL intensity of LaAlO_3_:2%Mn^4+^ phosphor. In particular, the PL emission of LaAlO_3_:2%Mn^4+^,2%Na^+^, LaAlO_3_:2%Mn^4+^,2%Ca^2+^, LaAlO_3_:2%Mn^4+^,2%Ba^2+^, and LaAlO_3_:2%Mn^4+^,2%Sr^2+^ was several times stronger than that of LaAlO_3_:2%Mn^4+^ phosphor. It indicates that employing the charge compensation strategy with appropriate co-dopants such as Na^+^, Cl^−^, Ge^4+^, Ca^2+^, Sr^2+^ and Ba^2+^ ions helps to achieve the improvement of luminescence. Mn^4+^ ions and these R co-dopants were successfully incorporated into the LaAlO_3_ host compound.

**Figure 8:**
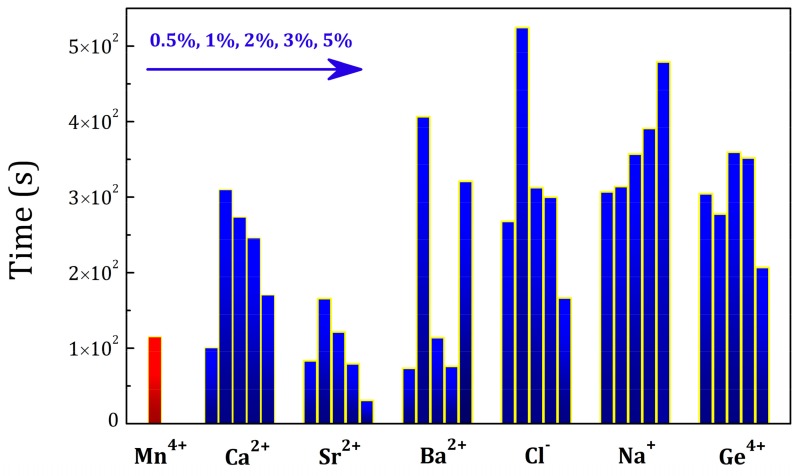
A comparison of the time until the intensity of afterglow luminescence drops to 5 × 10^−4^ mW/sr/m^2^.The concentration of Mn^4+^ ion is 0.5% in each case and the concentration of R is 0.5%, 1%, 2%, 3% and 5% respectively from left to right in each doping group (R = Ca^2+^, Sr^2+^, Ba^2+^, Cl^−^, Na^+^, Ge^4+^). The first column corresponds to LaAlO_3_:0.5%Mn^4+^ as an intensity and duration benchmark of persistent luminescence.

**Figure 9:**
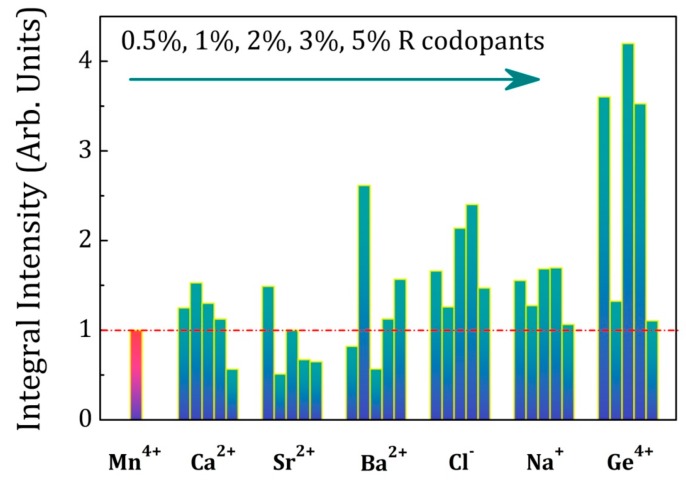
A map of integral intensity of photoluminescence spectra from Mn^4+^/R (R = Ca^2+^, Sr^2+^, Ba^2+^, Cl^−^, Na^+^, Ge^4+^) co-dopants in the wavelength from 600 nm to 800 nm. The concentration of Mn^4+^ ions is 0.5% and the concentration of R is 0.5%, 1%, 2%, 3% and 5% respectively from left to right in each doping group.

In order to optimize the properties of LaAlO_3_:Mn^4+^ phosphors with the charge compensation strategy, six groups of LaAlO_3_:Mn^4+^, R (R = Na^+^, Cl^−^, Ge^4+^, Ca^2+^, Sr^2+^, Ba^2+^) phosphors with different co-dopant concentrations were synthesized using the same conditions as discussed above, now using the optimum Mn^4+^ concentration of 0.5%. The concentration of R (R = Na^+^, Cl^−^, Ge^4+^, Ca^2+^, Sr^2+^, Ba^2+^) was0.5%, 1%, 2%, 3% and 5%. The persistent luminescence decay curves were measured after 5 min of irradiation with a Xenon arc lamp and the detailed persistent luminescence decay curves of LaAlO_3_:0.5%Mn^4+^,yR (R = Na^+^, Cl^−^, Ge^4+^, Ca^2+^, Sr^2+^, Ba^2+^; y = 0.5%, 1%, 2%, 3% and 5%) phosphors are shown respectively in Figures S5–S10. A comparison of the afterglow duration time of the six groups is illustrated in Figure 8, showing the time after excitation when the intensity of the afterglow luminescence drops to 5 × 10^−4^ mW/sr/m^2^. This benchmark intensity roughly corresponds to the same intensity as the 0.3 mcd/m^2^ value, used as a benchmark for visible persistent luminescence [3]. The first red column corresponds to LaAlO_3_:0.5%Mn^4+^ as an intensity and duration reference of persistent luminescence. The concentration of R (R = Ca^2+^, Sr^2+^, Ba^2+^, Cl^−^, Na^+^, Ge^4+^) is increasing from left to right in each doping group in Figure 8. The afterglow duration time is prolonged when increasing the concentration of co-dopants and decreases upon further increasing concentrations in the case of Ca^2+^, Sr^2+^, Cl^−^ co-dopants, similar to the Mn^4+^ doping behavior as discussed above in Figure 5. For other co-dopants, the trend turns abnormal due to the complex interactions between the traps and defects. All the co-dopants chosen in this research can help to improve the persistent luminescence and strengthen the afterglow duration time to different extent with various doping concentrations. Steady state photoluminescence spectra of the six groups of LaAlO_3_:Mn^4+^,R (R = Na^+^, Cl^−^, Ge^4+^, Ca^2+^, Sr^2+^, Ba^2+^) phosphors were measured at room temperature. For each group of LaAlO_3_:Mn^4+^,R phosphors with different co-dopant concentrations, the intensities of photoluminescence were enhanced to a different extent for certain doping concentrations and PL spectra are shown in Figures S11–S16. This also proves the feasibility of the charge compensation strategy for LaAlO_3_:Mn^4+^ phosphors. Figure 9 exhibits a map of the integrated intensity of photoluminescence from Mn^4+^ with R (R = Ca^2+^, Sr^2+^, Ba^2+^, Cl^−^, Na^+^, Ge^4+^) co-dopants. The integral intensity was calculated in the wavelength region from 600 nm to 800 nm. The concentration of Mn^4+^ ion was 0.5% in each LaAlO_3_ host and the concentration of R is 0.5%, 1%, 2%, 3% and 5% respectively from left to right in each doping group as illustrated in Figure 9. In order to compare the influence among the different co-dopants with the different R concentrations, the integral intensity of LaAlO_3_:0.5%Mn^4+^ emission spectrum was normalized to 1 as a benchmark of emission intensity. It indicates that co-doping with Ge^4+^, Na^+^, Cl^−^, Ba^2+^, Sr^2+^ and Ca^2+^ is beneficial for the enhancement of the PL intensity. Upon doping only one type of dopant, like Mn^4+^ ions, the PL and afterglow properties of LaAlO_3_:Mn^4+^ phosphors have a regular trend when increasing the concentrations of Mn^4+^ ions from 0.1% to 5% as shown in Figure 4 and Figure 5. However, upon doping two kinds of dopants with the phosphor formula ‘LaAlO_3_:Mn^4+^,R’, the possible mechanism and interactions between the traps and defects turns complicated, resulting in an anomalous performance with different concentrations of dopants and co-dopants. The duration and PL intensity of the Mn^4+^ emission may not simply change monotonously when co-doping another R ions as it is shown in Figure 8 and Figure 9. When co-doping with different ions, even isovalent Ge^4+^, it is possible that the optimum Mn dopant concentration is changed, again leading to a more complex relation between co-dopant concentration and performance. In view of the improved performance of Mn^4+^-activated LaAlO_3_ phosphor, co-dopants chosen in this research can lead to a 2-fold to 4-fold increase of afterglow time and PL intensity with different doping concentrations. Based on this preliminary screening of the performance of different co-dopants, a more in-depth investigation of the effects of co-doping on afterglow performance will be conducted on selected co-dopants, using temperature dependent charging and afterglow experiments and thermoluminescence measurements.

PL intensities and afterglow duration of the LaAlO_3_:Mn^4+^ phosphors are determined by the competition between the quantity of the effective Mn^4+^ ions (defect density of Mn^4+^ ions) in the phosphors and the interaction among the Mn^4+^ ions (Mn^4+^-Mn^4+^ pairs). We believe that more isolated Mn^4+^ ions in the phosphors enhance the light emission and more traps and defects strengthen the afterglow duration. However, the formation and interaction from Mn^4+^-Mn^4+^ pairs, which are inevitably formed at high annealing temperature as reported in some other papers [43,46], will decrease both defect density and the number of effective Mn^4+^ ions, resulting in quenching the emission and shorten afterglow duration. From the Figure 8 and Figure 9, both the photoluminescence and persistent luminescence can be enhanced to a certain extent when employing the appropriate co-dopants such as Na^+^, Cl^−^, Ca^2+^, Ba^2+^, Sr^2+^ and Ge^4+^ ions. As for the improvement of persistent luminescence, two kinds of active centers are involved in persistent luminescence, namely traps and emitters. Traps originate from lattice defects or co-dopants in the phosphor and emitters release light in the region of interest. It is known that Mn^4+^ ion can act as both the trapping center and the emitting center in the perovskite LaAlO_3_ host [19]. The afterglow duration thus relies on the effective defect density of Mn^4+^ ions as trapping centers. In addition, Mn^4+^ trapping centers have a complex dependency on the concentration of incorporated manganese ions and Mn^4+^-Mn^4+^ pairs [43]. Mn^4+^-Mn^4+^ pairs decrease the effective defect density of Mn^4+^ ions and weaken the persistent luminescence. The afterglow of this material is constrained by the formation of Mn^4+^-Mn^4+^ pairs in the host, resulting in a lower effective defect density. The challenge in the perovskite LaAlO_3_ host is to incorporate more Mn^4+^ ions as traps and avoid Mn^4+^-Mn^4+^ pairs. Charge-compensating co-dopants help to impede the formation of Mn^4+^-Mn^4+^ pairs. In this material, the effective incorporation and increasing defect density are strongly improved by co-doping with Ge^4+^ or ions with a lower valence state for charge compensation, such as divalent cations (Ca^2+^, Ba^2+^ or Sr^2+^ ions), monovalent cations (Na^+^ ions) or ions in their negative valence (Cl^−^ ions). Thus, the enrichment of the effective defect density prolongs the persistent luminescence. As for the photoluminescence enhancement, the origin of this phenomenon is widely understood and explained by the charge compensation mechanism. The photoluminescence intensity is determined by the complex competition between the effective amount of Mn^4+^ ions and quantity of the Mn^4+^-Mn^4+^ pairs in LaAlO_3_:Mn^4+^ phosphors. It is believed that more effective Mn^4+^ ions increase the PL intensity while the Mn^4+^-Mn^4+^ pairs quench the emission. The interaction of Mn^4+^-Mn^4+^ pairs is related to the Mn^4+^-Mn^4+^ distance [43]. Substitution of 6-fold coordinated octahedral Al^3+^ ion or 12-fold coordinated central La^3+^ ion with a lower charge state is supposed to create a negative local charge, which improves the efficiency of Mn^4+^ ions to replace Al^3+^ sites, thus increasing the effective amount of Mn^4+^ ions. Furthermore, the incorporation with lower valence state ions guarantees the charge equilibrium, which restrains the formation of Mn^4+^-Mn^4+^ pairs leading to the enhancement of PL intensity [44]. That is the case for divalent cations (Ca^2+^, Ba^2+^ or Sr^2+^ ions), monovalent cations (Na^+^ ions) and negative valence ions (Cl^−^ ions). Tetravalent cations such as Ge^4+^ ions are competitive with the substitution by Mn^4+^ due to the same valence state. However, the co-dopant Ge^4+^ ions may enrich the defect density and play an effective role in decreasing the formation and interaction of Mn^4+^-Mn^4+^ pairs, resulting as a compensator to Mn^4+^-Mn^4+^ pairs and enhancing the PL intensity [19,43]. In the case of the perovskite LaAlO_3_ host, the compensation effect of co-doping Mn^4+^/Ge^4+^ ions is found to occupy a predominant position.

## 3. Materials and Methods

All the raw chemicals were analytical grade, used without further purification. The precursors were La_2_O_3_ (Sigma Aldrich, Saint Louis, MO, USA, 99.99%), Al_2_O_3_ (Fluka, Schwerte, Germany, 99.5%), MnO_2_ (Alfa Aesar, Karlsruhe, Germany, 99.997%), NH_4_Cl (Alfa Aesar, 99.999%), Li_2_CO_3_ (Alfa Aesar, 99.998%), Na_2_CO_3_ (Alfa Aesar, 99.95%), MgO (Alfa Aesar, 99.95%), CaCO_3_ (Alfa Aesar, 99.95%), SrCO_3_ (Alfa Aesar, 99.99%), BaCO_3_ (Alfa Aesar, 99.95%), SiO_2_ (Alfa Aesar, 99.5%), GeO_2_ (Alfa Aesar, 99.999%), TiO_2_ (Alfa Aesar, 99.995%), ZrO_2_ (Alfa Aesar, 99.978%). The concentrations of dopants were chosen as follows: LaAlO_3_:xMn^4+^ (x = 0.1%, 0.2%, 0.5%, 1%, 2% and 5%); LaAlO_3_:2%Mn^4+^,2%R; LaAlO_3_:0.5%Mn^4+^,yR (y = 0.5%, 1%, 2%, 3% and 5%). The molar % is defined with respect to one mole of a host phosphor chemical formula. The appropriate stoichiometric number of precursors were weighed and manually ground in an agate mortar. Subsequently, the starting materials were mixed with ethanol and put in a ZrO_2_ grinding jar. Grinding was performed in a Retsch PM 100 Planetary ball mill for 6 h to reduce the particle size. After evaporating the remaining ethanol, repeated grinding was performed in an agate mortar to improve the mixing homogeneity of the precursors. The phosphors of LaAlO_3_:Mn^4+^ and a series of LaAlO_3_:Mn^4+^,R (R = Cl^−^, Li^+^, Na^+^, Mg^2+^, Ca^2+^, Sr^2+^, Ba^2+^, Si^4+^, Ge^4+^, Ti^4+^, Zr^4+^) were synthesized through a traditional high-temperature solid-state reaction method, testing various temperatures between 1350 °C and 1650 °C, for 6 h in air. The employed heating rate was 300 °C/h using a tube furnace (ETF30-50/18-S furnace, ENTECH, Ängelholm, Sweden). All samples were allowed to cool naturally inside the tube furnace. The sintered samples were well ground again. To compare the luminescence properties and afterglow intensity, all the phosphors were synthesized under the same experimental conditions.

The crystal structures of LaAlO_3_:Mn^4+^ and LaAlO_3_:Mn^4+^, R were characterized by Powder X-ray diffraction (XRD) (Bruker, Leiderdorp, The Nederlands). Crystallographic phases of the obtained powders were verified on a Siemens D5000 diffractometer (40 kV, 40 mA, Bruker) using Cu Kα1 radiation (λ = 0.154 nm). The XRD data were collected in the range 2θ from 10° to 80° at room temperature. A comparison of the obtained XRD patterns with the reference pattern (No. 00-031-0022) was made to check the phase purity.

Steady state photoluminescence excitation and emission spectra were measured using an Edinburgh FS920 (Edinburgh Instruments Ltd., Livingston, UK) fluorescence spectrometer with a monochromated 450 W Xe-arc lamp as the excitation source.

The powder was put into a metal disc with a diameter of 12.5 mm and persistent luminescence was measured with a photosensor amplifier (C9329, Hamamatsu, Japan) and a Centronics OSD100-5T (Centronic Ltd., Croydon, UK) silicon photodiode. The afterglow decay profiles were then calibrated to the absolute radiance (in unit of mW/sr/m^2^), since the usual units of luminance, cd/m^2^, are not relevant for near-infrared emitting phosphors [3]. All persistent luminescent decay curves of LaAlO_3_ samples were recorded at room temperature after excitation for 5 min by the light of an unfiltered Xenon arc lamp at an intensity of 1000 lux.

## 4. Conclusions

In summary, a series of novel near-infrared emitting persistent luminescent phosphors LaAlO_3_:Mn^4+^ (LAO:Mn^4+^) were synthesized by a convenient high-temperature solid-state reaction in air. Various kinds of co-dopants with Mn^4+^ ions were optimized and incorporated in different concentrations. Impressively, co-dopants such as Cl^−^, Na^+^, Ca^2+^, Sr^2+^, Ba^2+^ and Ge^4+^ ions were all found to be beneficial for improving the LaAlO_3_:Mn^4+^ luminescence and afterglow intensity. The charge compensation strategies for LaAlO_3_:Mn^4+^ phosphors were systematically discussed. Employing this charge compensation strategy is believed to open up new avenues for the exploration of more promising near-infrared emitting long persistent phosphors for medical imaging.

## Supplementary Materials

The following are available online at www.mdpi.com/1996-1944/10/12/1422/s1, Figure S1: XRD pattern of LaAlO_3_ synthesized through a solid-state reaction method, Figure S2: Photoluminescence (PL) spectra of LaAlO_3_:2%Mn^4+^,2%Li^+^, LaAlO_3_:2%Mn^4+^,2%Na^+^, and LaAlO_3_:2%Mn^4+^,2%Cl^−^ phosphors, Figure S3: Photoluminescence (PL) spectra of LaAlO_3_:2%Mn^4+^,2%Ge^4+^, LaAlO_3_:2%Mn^4+^,2%Si^4+^, LaAlO_3_:2%Mn^4+^,2%Ti^4+^, and LaAlO_3_:2%Mn^4+^,2%Zr^4+^ phosphors, Figure S4: Photoluminescence (PL) spectra of LaAlO_3_:2%Mn^4+^,2%Ba^2+^, LaAlO_3_:2%Mn^4+^,2%Ca^2+^, LaAlO_3_:2%Mn^4+^,2%Mg^2+^,and LaAlO_3_:2%Mn^4+^,2%Sr^2+^ phosphors, Figure S5: Persistent luminescence decay curves of LaAlO_3_:0.5%Mn^4+^,ySr^2+^ (y = 0.5%, 1%, 2%, 3%, and 5%) phosphors after 5 min of irradiation with a Xenon arc lamp, Figure S6: Persistent luminescence decay curves of LaAlO_3_:0.5%Mn^4+^,yGe^4+^ (y = 0.5%, 1%, 2%, 3%, and 5%) phosphors after 5 min of irradiation with a Xenon arc lamp, Figure S7: Persistent luminescence decay curves of LaAlO_3_:0.5%Mn^4+^,yCa^2+^ (y = 0.5%, 1%, 2%, 3%, and 5%) phosphors after 5 min of irradiation with a Xenon arc lamp, Figure S8: Persistent luminescence decay curves of LaAlO_3_:0.5%Mn^4+^,yBa^2+^ (y = 0.5%, 1%, 2%, 3%, and 5%) phosphors after 5 min of irradiation with a Xenon arc lamp, Figure S9: Persistent luminescence decay curves of LaAlO_3_:0.5%Mn^4+^,yCl^−^ (y = 0.5%, 1%, 2%, 3%, and 5%) phosphors after 5 min of irradiation with a Xenon arc lamp, Figure S10: Persistent luminescence decay curves of LaAlO_3_:0.5%Mn^4+^,yNa^+^ (y = 0.5%, 1%, 2%, 3%, and 5%) phosphors after 5 min of irradiation with a Xenon arc lamp, Figure S11: Photoluminescence (PL) spectra of LaAlO_3_:0.5%Mn^4+^,0.5%Ge^4+^, LaAlO_3_:0.5%Mn^4+^,1%Ge^4+^, LaAlO_3_:0.5%Mn^4+^,2%Ge^4+^, LaAlO_3_:0.5%Mn^4+^,3%Ge^4+^ and LaAlO_3_:0.5%Mn^4+^,5%Ge^4+^ phosphors, Figure S12: Photoluminescence (PL) spectra of LaAlO_3_:0.5%Mn^4+^,0.5%Ba^2+^, LaAlO_3_:0.5%Mn^4+^,1%Ba^2+^, LaAlO_3_:0.5%Mn^4+^,2%Ba^2+^, LaAlO_3_:0.5%Mn^4+^,3%Ba^2+^ and LaAlO_3_:0.5%Mn^4+^,5%Ba^2+^ phosphors, Figure S13: Photoluminescence (PL) spectra of LaAlO_3_:0.5%Mn^4+^,0.5%Sr^2+^, LaAlO_3_:0.5%Mn^4+^,1%Sr^2+^, LaAlO_3_:0.5%Mn^4+^,2%Sr^2+^, LaAlO_3_:0.5%Mn^4+^,3%Sr^2+^ and LaAlO_3_:0.5%Mn^4+^,5%Sr^2+^ phosphors, Figure S14: Photoluminescence (PL) spectra of LaAlO_3_:0.5%Mn^4+^,0.5%Ca^2+^, LaAlO_3_:0.5%Mn^4+^,1%Ca^2+^, LaAlO_3_:0.5%Mn^4+^,2%Ca^2+^, LaAlO_3_:0.5%Mn^4+^,3%Ca^2+^ and LaAlO_3_:0.5%Mn^4+^,5%Ca^2+^ phosphors, Figure S15: Photoluminescence (PL) spectra of LaAlO_3_:0.5%Mn^4+^,0.5%Na^+^, LaAlO_3_:0.5%Mn^4+^,1%Na^+^, LaAlO_3_:0.5%Mn^4+^,2%Na^+^, LaAlO_3_:0.5%Mn^4+^,3%Na^+^ and LaAlO_3_:0.5%Mn^4+^,5%Na^+^ phosphors, Figure S16: Photoluminescence (PL) spectra of LaAlO_3_:0.5%Mn^4+^,0.5%Cl^−^, LaAlO_3_:0.5%Mn^4+^,1%Cl^−^, LaAlO_3_:0.5%Mn^4+^,2%Cl^−^, LaAlO_3_:0.5%Mn^4+^,3%Cl^−^ and LaAlO_3_:0.5%Mn^4+^,5%Cl^−^ phosphors, Table S1: Ionic radius of some common dopant cations for the substitution on Al^3+^ site, Table S2: Ionic radius of some common dopant cations for the substitution on La^3+^ site.
