# Supplementary material for: LaAlO3:Mn4+ as Near-Infrared Emitting Persistent Luminescence Phosphor for Medical Imaging: A Charge Compensation Study
Source: Materials (Basel). 2017 Dec 12;10(12):1422. doi: 10.3390/ma10121422 (PMC5744357; doi:10.3390/ma10121422)
Supplement: Supplementary file 1 [file materials-10-01422-s001.pdf]

nanomaterials

Article-Supporting Information

$LaAlO_3:Mn^{4+}$ as near-infrared emitting persistent luminescence phosphor for medical imaging: A charge compensation study

Jiaren Du [1], Olivier Q. De Clercq [1], Katleen Korthout [1] and Dirk Poelman [1,*]

[1] LumiLab, Department of Solid State Sciences, Ghent University, Krijgslaan 281-S1, Ghent, Belgium; Jiaren.Du@ugent.be; Olivier.DeClercq@ugent.be; Katleen.Korthout@ugent.be
* Correspondence: Dirk.Poelman@ugent.be; Tel.: +32-9264-4367

Figure S1-S16; Table S1,S2

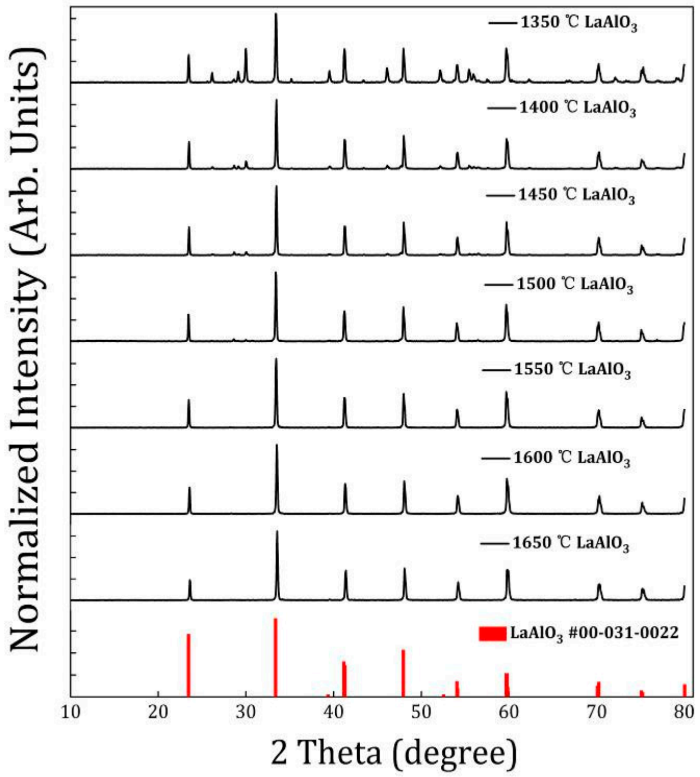

Figure S1. XRD pattern of $LaAlO_3$ synthesized through a solid-state reaction method. The synthesis temperature is indicated next to the diffractograms. The intensities of the XRD patterns are normalized to arbitrary units [0, 1].

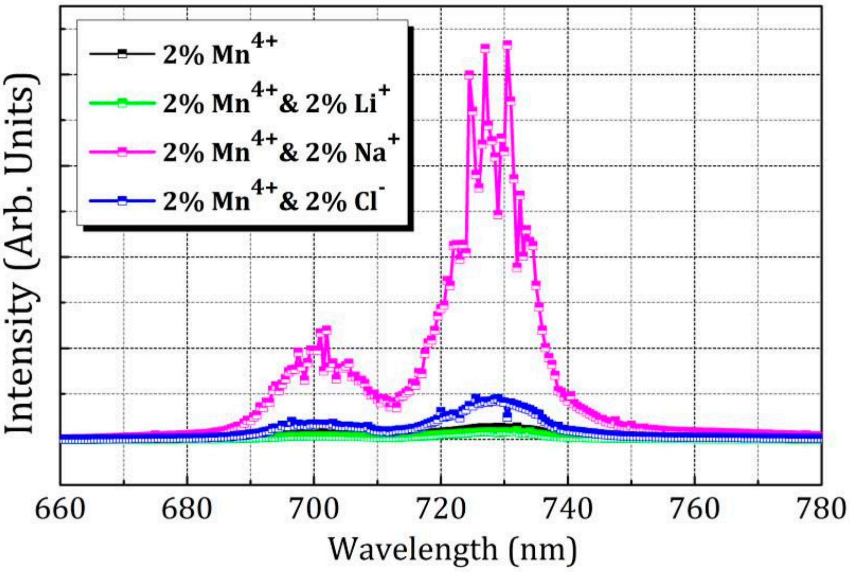

Figure S2. Photoluminescence (PL) spectra of LaAlO$_3$:2%Mn^{4+},2%Li$^+$, LaAlO$_3$:2%Mn^{4+},2%Na$^+$, and LaAlO$_3$:2%Mn^{4+},2%Cl$^-$ phosphors. All the PL spectra (λ_{ex}= 335 nm) are in the range 660-780 nm and PL spectrum of LaAlO$_3$:2%Mn^{4+} phosphor is shown in black for comparison.

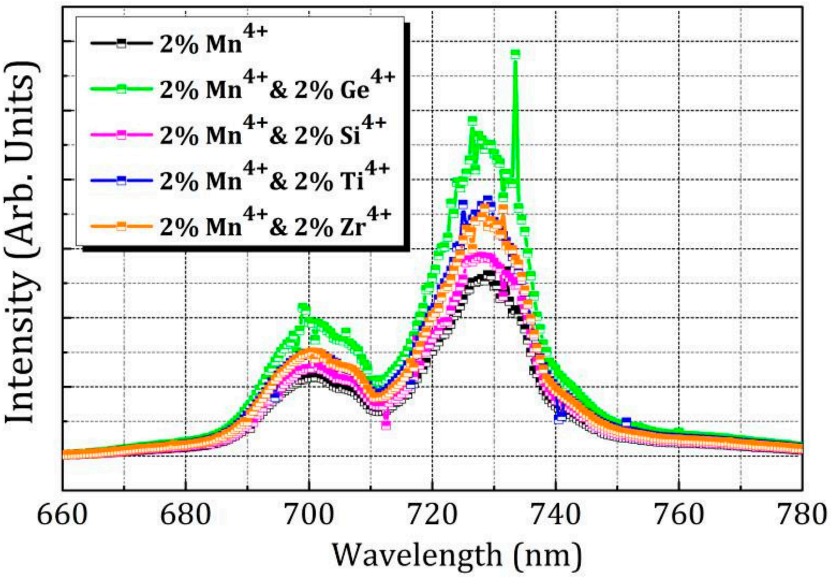

Figure S3. Photoluminescence (PL) spectra of LaAlO$_3$:2%Mn^{4+},2%Ge^{4+}, LaAlO$_3$:2%Mn^{4+},2%Si^{4+}, LaAlO$_3$:2%Mn^{4+},2%Ti^{4+},and LaAlO$_3$:2%Mn^{4+},2%Zr^{4+} phosphors. All the PL spectra (λ_{ex}= 335 nm) are in the range 660-780 nm and PL spectrum of LaAlO$_3$: 2%Mn^{4+} phosphor is shown in black for comparison.

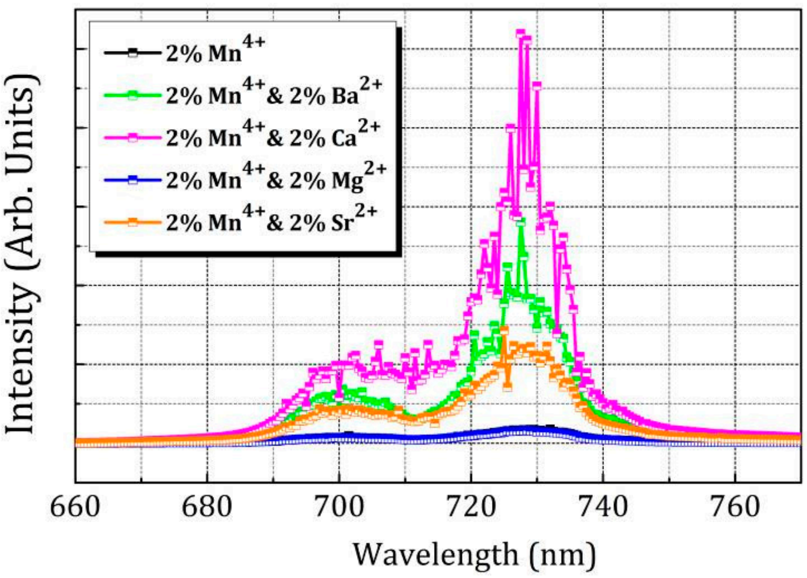

Figure S4. Photoluminescence (PL) spectra of $LaAlO_3$:2%Mn^{4+},2%Ba^{2+}, $LaAlO_3$:2%Mn^{4+},2%Ca^{2+}, $LaAlO_3$:2%Mn^{4+},2%Mg^{2+},and $LaAlO_3$:2%Mn^{4+},2%Sr^{2+} phosphors. All the PL spectra (λ_{ex}= 335 nm) are in the range 660-780 nm and PL spectrum of $LaAlO_3$:2%Mn^{4+} phosphor is shown in black for comparison.

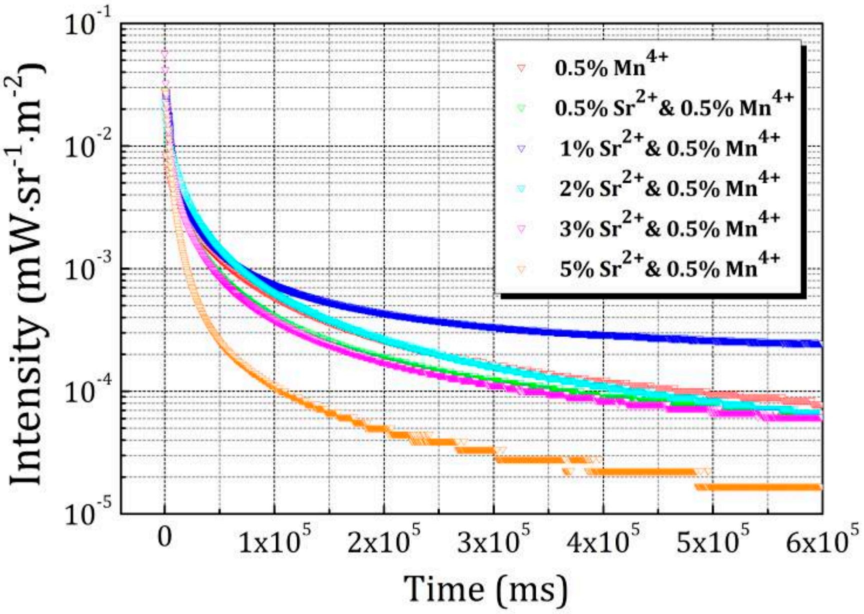

Figure S5. Persistent luminescence decay curves of $LaAlO_3$:0.5%Mn^{4+},ySr^{2+} (y= 0.5%, 1%, 2%, 3%, and 5%) phosphors after 5 min of irradiation with a Xenon arc lamp. The red curve corresponds to $LaAlO_3$:0.5%Mn^{4+} as an intensity benchmark of persistent luminescence.

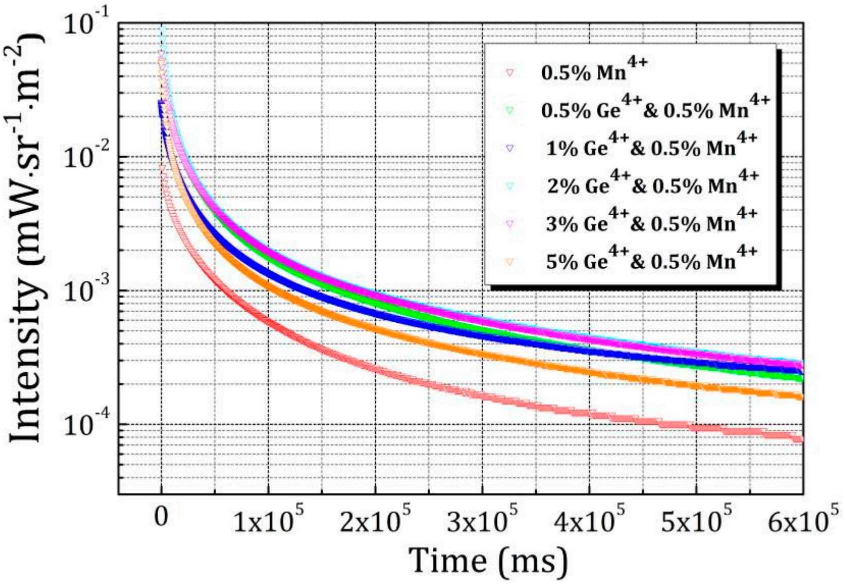

Figure S6. Persistent luminescence decay curves of $LaAlO_3$:0.5%Mn^{4+},yGe^{4+} (y = 0.5%, 1%, 2%, 3%, and 5%) phosphors after 5 min of irradiation with a Xenon arc lamp. The red curve corresponds to $LaAlO_3$:0.5%Mn^{4+} as an intensity benchmark of persistent luminescence.

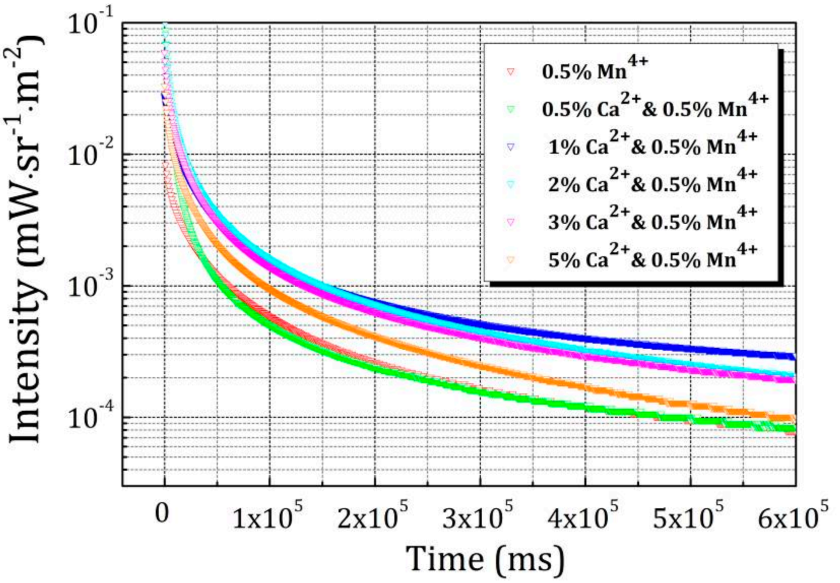

Figure S7. Persistent luminescence decay curves of $LaAlO_3$:0.5%Mn^{4+},yCa^{2+} (y = 0.5%, 1%, 2%, 3%, and 5%) phosphors after 5 min of irradiation with a Xenon arc lamp. The red curve corresponds to $LaAlO_3$: 0.5%Mn^{4+} as an intensity benchmark of persistent luminescence.

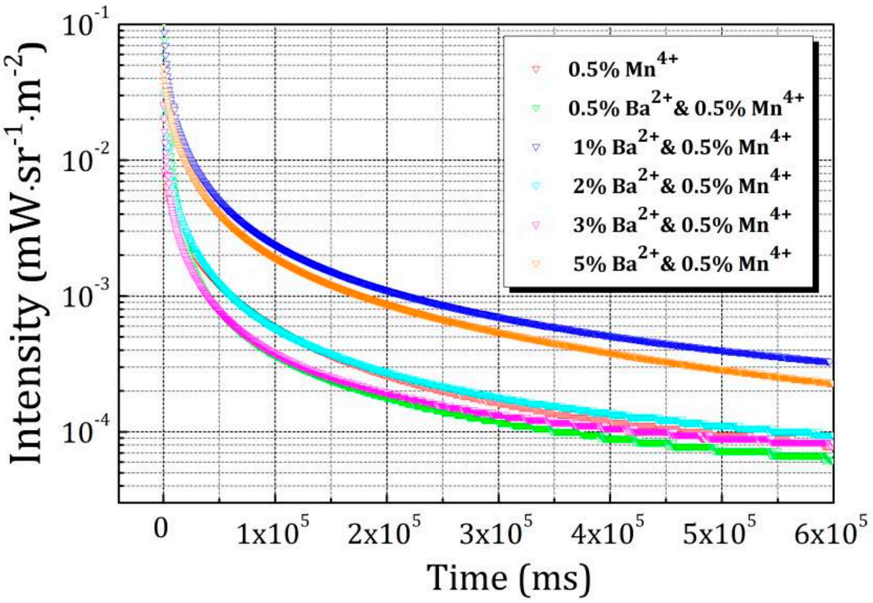

Figure S8. Persistent luminescence decay curves of $LaAlO_3$:0.5%Mn^{4+},yBa^{2+} (y = 0.5%, 1%, 2%, 3%, and 5%) phosphors after 5 min of irradiation with a Xenon arc lamp. The red curve corresponds to $LaAlO_3$: 0.5%Mn^{4+} as an intensity benchmark of persistent luminescence.

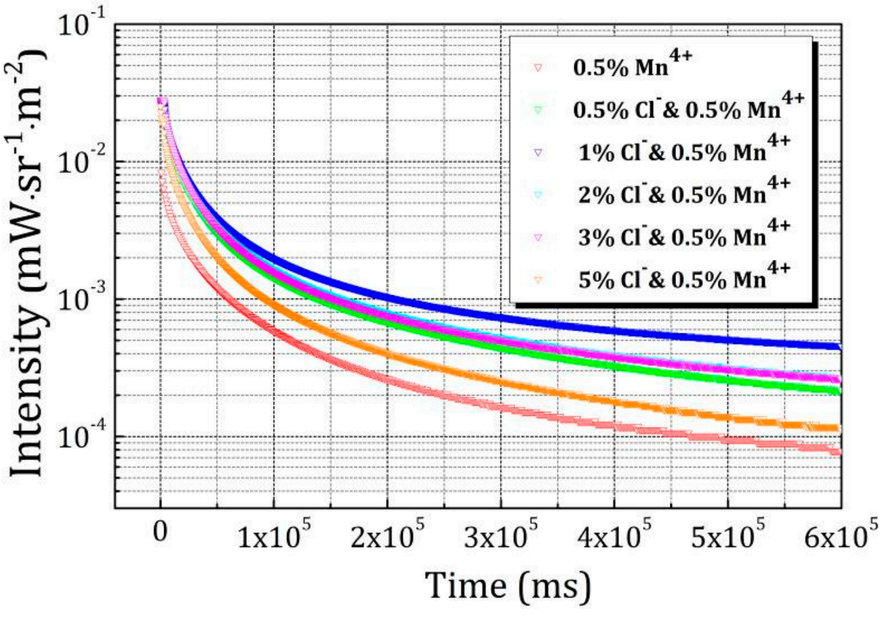

Figure S9. Persistent luminescence decay curves of $LaAlO_3$:0.5%Mn^{4+},yCl^- (y = 0.5%, 1%, 2%, 3%, and 5%) phosphors after 5 min of irradiation with a Xenon arc lamp. The red curve corresponds to $LaAlO_3$:0.5%Mn^{4+} as an intensity benchmark of persistent luminescence.

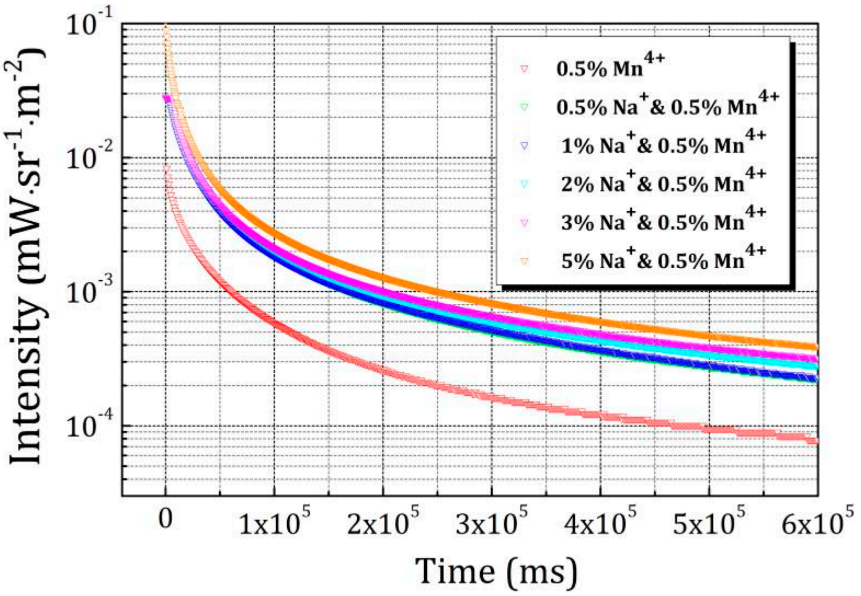

Figure S10. Persistent luminescence decay curves of $LaAlO_3$:0.5%Mn^{4+},yNa^{+} (y = 0.5%, 1%, 2%, 3%, and 5%) phosphors after 5 min of irradiation with a Xenon arc lamp. The red curve corresponds to $LaAlO_3$:0.5%Mn^{4+} as an intensity benchmark of persistent luminescence.

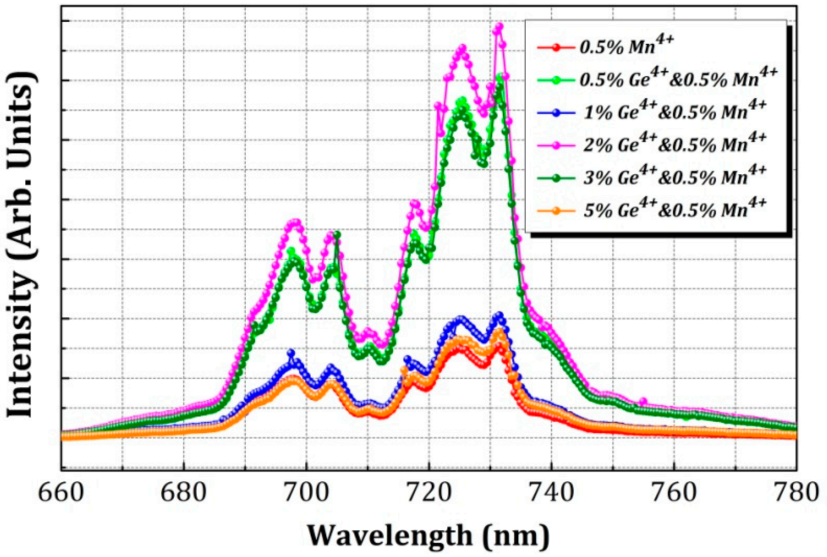

Figure S11. Photoluminescence (PL) spectra of $LaAlO_3$:0.5%Mn^{4+},0.5%Ge^{4+}, $LaAlO_3$:0.5%Mn^{4+},1%Ge^{4+}, $LaAlO_3$:0.5%Mn^{4+},2%Ge^{4+}, $LaAlO_3$:0.5%Mn^{4+},3%Ge^{4+} and $LaAlO_3$:0.5%Mn^{4+},5%Ge^{4+} phosphors. All the PL spectra (λ_{ex}= 335 nm) are in the range 660-780 nm and PL spectrum of $LaAlO_3$:0.5%Mn^{4+} phosphor is shown in red for comparison.

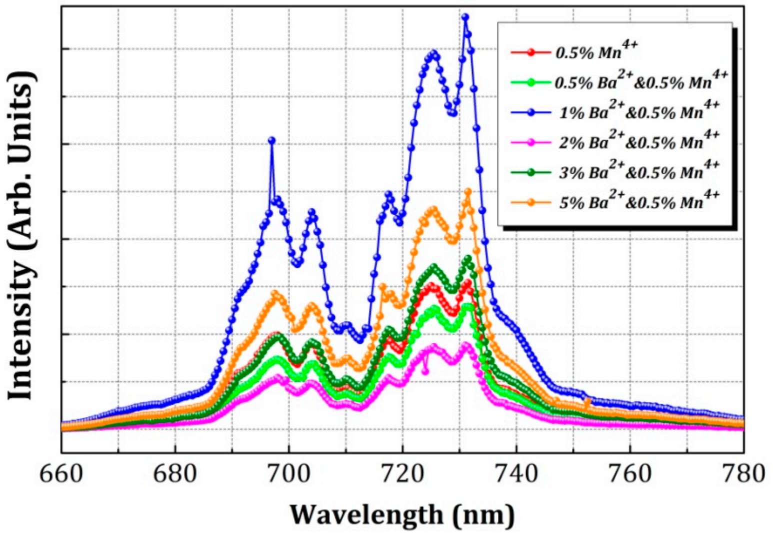

Figure S12. Photoluminescence (PL) spectra of $LaAlO_3$:0.5%Mn^{4+},0.5%Ba^{2+}, $LaAlO_3$:0.5%Mn^{4+},1%Ba^{2+}, $LaAlO_3$:0.5%Mn^{4+},2%Ba^{2+}, $LaAlO_3$:0.5%Mn^{4+},3%Ba^{2+} and $LaAlO_3$:0.5%Mn^{4+},5%Ba^{2+} phosphors. All the PL spectra (λ_{ex}= 335 nm) are in the range 660-780 nm and PL spectrum of $LaAlO_3$:0.5%Mn^{4+} phosphor is in red for comparison.

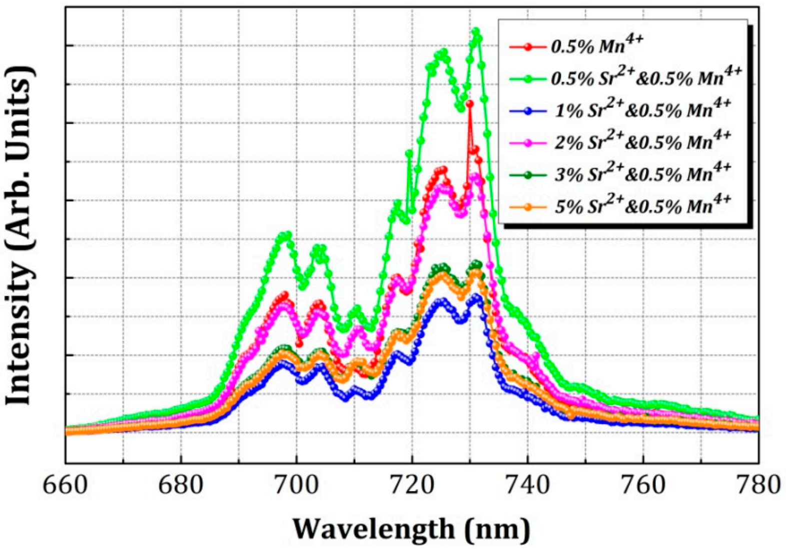

Figure S13. Photoluminescence (PL) spectra of $LaAlO_3$:0.5%Mn^{4+},0.5%Sr^{2+}, $LaAlO_3$:0.5%Mn^{4+},1%Sr^{2+}, $LaAlO_3$:0.5%Mn^{4+},2%Sr^{2+}, $LaAlO_3$:0.5%Mn^{4+},3%Sr^{2+} and $LaAlO_3$:0.5%Mn^{4+},5%Sr^{2+} phosphors. All the PL spectra (λ_{ex}= 335 nm) are in the range 660-780 nm and PL spectrum of $LaAlO_3$:0.5%Mn^{4+} phosphor is in red for comparison.

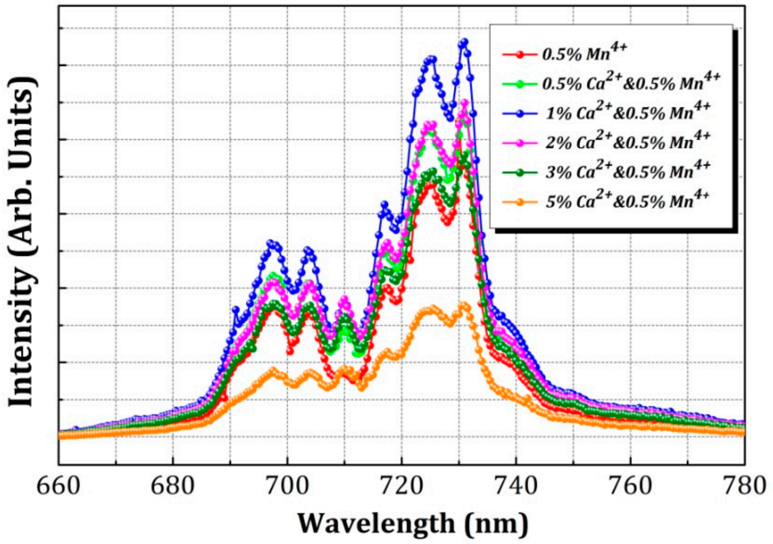

Figure S14. Photoluminescence (PL) spectra of $LaAlO_3$:0.5%Mn^{4+},0.5%Ca^{2+}, $LaAlO_3$:0.5%Mn^{4+},1%Ca^{2+}, $LaAlO_3$:0.5%Mn^{4+},2%Ca^{2+}, $LaAlO_3$:0.5%Mn^{4+},3%Ca^{2+} and $LaAlO_3$:0.5%Mn^{4+},5%Ca^{2+} phosphors. All the PL spectra (λ_{ex}= 335 nm) are in the range 660-780 nm and PL spectrum of $LaAlO_3$:0.5%Mn^{4+} phosphor is in red for comparison.

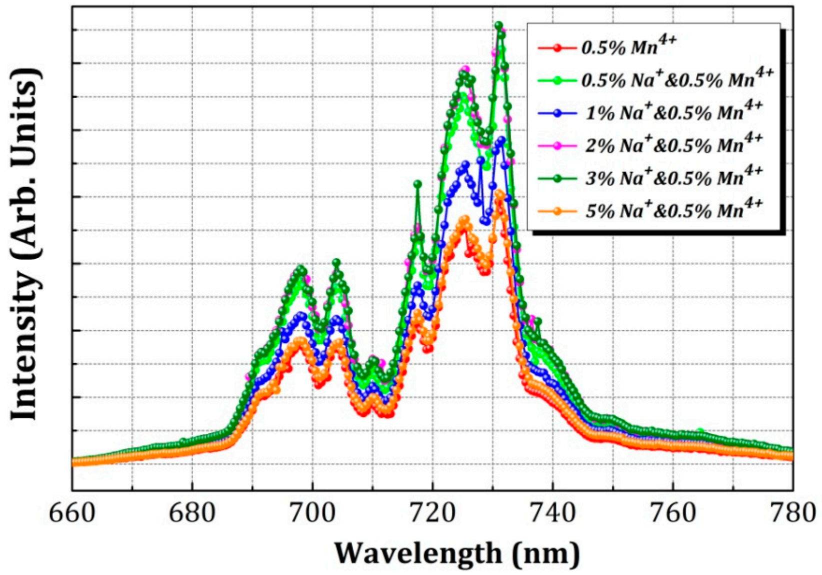

Figure S15. Photoluminescence (PL) spectra of $LaAlO_3$:0.5%Mn^{4+},0.5%Na^{+}, $LaAlO_3$:0.5%Mn^{4+},1%Na^{+}, $LaAlO_3$:0.5%Mn^{4+},2%Na^{+}, $LaAlO_3$:0.5%Mn^{4+},3%Na^{+} and $LaAlO_3$:0.5%Mn^{4+},5%Na^{+} phosphors. All the PL spectra (λ_{ex}= 335 nm) are in the range 660-780 nm and PL spectrum of $LaAlO_3$:0.5%Mn^{4+} phosphor is in red for comparison.

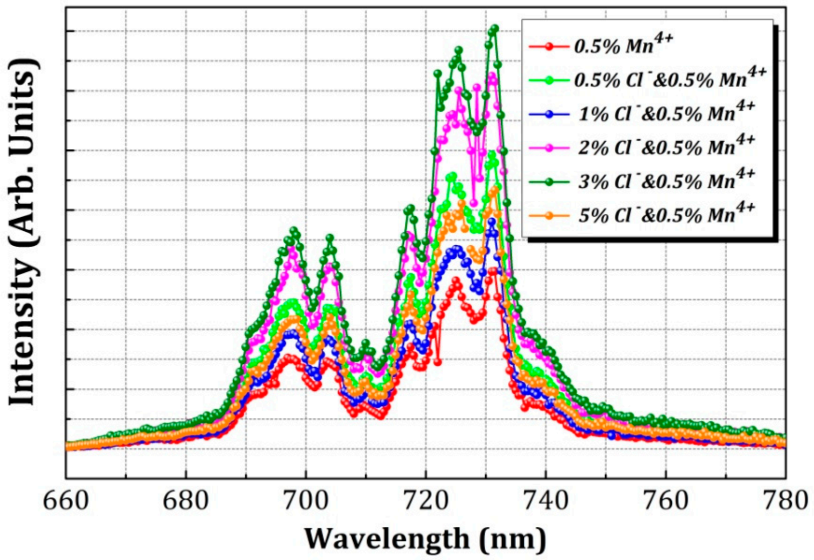

Figure S16. Photoluminescence (PL) spectra of $LaAlO_3$:0.5%Mn^{4+},0.5%Cl^-, $LaAlO_3$:0.5%Mn^{4+},1%Cl^-, $LaAlO_3$:0.5%Mn^{4+},2%Cl^-, $LaAlO_3$:0.5%Mn^{4+},3%Cl^- and $LaAlO_3$:0.5%Mn^{4+},5%Cl^- phosphors. All the PL spectra (λ_{ex}= 335 nm) are in the range 660-780 nm and PL spectrum of $LaAlO_3$:0.5%Mn^{4+} phosphor is in red for comparison.

Table S1. Ionic radius of some common dopant cations for the substitution on Al^{3+} site

Dopant Cations	Coordination Number (Substitution on Octahedral Al^{3+} Site)	Ionic Radius (pm)	$\lvert R_{DC^{2+}}-R_{Al^{3+}}\rvert / R_{Al^{3+}}$ (%)
Li^+	Ⅵ	76	42.05
Na^+	Ⅵ	102	90.65
K^+	Ⅵ	138	157.94
Rb^+	Ⅵ	152	184.11
Cs^+	Ⅵ	167	212.14
Ag^+	Ⅵ	115	114.95
Au^+	Ⅵ	137	156.07
Be^{2+}	Ⅵ	45	15.88
Mg^{2+}	Ⅵ	72	34.57
Ca^{2+}	Ⅵ	100	86.91
Sr^{2+}	Ⅵ	118	120.56
Ba^{2+}	Ⅵ	135	152.33
Cu^{2+}	Ⅵ	73	36.44
Zn^{2+}	Ⅵ	74	38.31
Cd^{2+}	Ⅵ	95	77.57
Hg^{2+}	Ⅵ	102	90.65
Sc^{3+}	Ⅵ	74.5	39.25
Y^{3+}	Ⅵ	90	68.22
B^{3+}	Ⅵ	27	49.53

Ga^{3+}	VI	62	15.88
In^{3+}	VI	80	49.53
Ti^{4+}	VI	60.5	13.08
Zr^{4+}	VI	72	34.57
Mn^{4+}	VI	53	0.93
Si^{4+}	VI	40	25.23
Ge^{4+}	VI	53	0.93
Sn^{4+}	VI	69	28.97

Table S2. Ionic radius of some common dopant cations for the substitution on La^{3+} site

Dopant Cations	Coordination Number (Substitution on La^{3+} Site)	Ionic Radius (pm)	$\lvert R_{DC^{2+}}-R_{La^{3+}}\rvert / R_{La^{3+}}$ (%)
Na^{+}	XII	139	2.20
K^{+}	XII	164	20.58
Rb^{+}	XII	172	26.47
Cs^{+}	XII	188	38.23
Ca^{2+}	XII	134	1.47
Sr^{2+}	XII	144	5.88
Ba^{2+}	XII	161	18.38
Cd^{2+}	XII	131	3.67
